# Acute Respiratory Failure as the First Manifestation of Antisynthetase Syndrome

**Published:** 2017

**Authors:** Sonia Toujani, Amani Ben Mansour, Meriem Mjid, Abir Hedhli, Jouda Cherif, Yassine Ouahchy, Majed Beji

**Affiliations:** Department of Respiratory, Research Unit 12SP06, Faculty of Medicine of Tunis, El Manar Tunis University, Rabta hospital, Tunisia.

**Keywords:** Antisynthetase syndrome, Respiratory failure, Interstitial lung disease

## Abstract

We report the case of a 40-year-old man with acute respiratory failure syndrome that later proved to be an initial manifestation of antisynthetase syndrome. The diagnosis of this rare combination of a connective tissue disease and an acute respiratory failure is difficult in a previously asymptomatic patient. Early diagnosis and immunosuppressive therapy started precociously prevented the disease progression and resulted in a good outcome.

## INTRODUCTION

Antisynthetase syndrome (ASS), first described as a heterogeneous connective tissue disease, is characterized as inflammatory myositis associated with fever, arthritis, Raynaud’s phenomenon, mechanic’s hands, and interstitial lung disease (ILD) with the presence of anti- RNA synthetase antibodies (ARS) (1). The most common anti-ARS antibody is anti- Jo-1. However, the combination of these findings is not always present in all patients. Diagnostic criteria of ASS requires the presence of any one of the several antisynthetase autoantibodies that target tRNA associated with one or more of the conditions, such as ILD, polymyositis, arthritis, unexplained persistent fever, Raynaud phenomenon, and mechanic’s hands (2,3). The most prevalent ASS manifestation associated with ARS is ILD. Moreover, ILD represents a major cause of morbidity and mortality in ASS (4, 5). Severe respiratory failure as the presenting feature of ILD associated with ASS is extremely rare (6). This is a recent case of a patient presenting with acute respiratory diagnosed as ASS.

## CASE SUMMARIES

A 40 year-old man, with a history of smoking (30 pack-years), was admitted to the pulmonology department for breathlessness, weakness, fever, and productive cough with rapid deterioration of respiratory conditions. He did not report any other symptoms and had been in good health until the last 3 weeks. The physical examination revealed the following: body temperature 38°C, respiratory rate 34 breaths/minute, blood pressure 120/75 mmHg, pulse rate 84 beats/minute, and oxygen saturation 85% on room air. Crackles were heard at the base of the lungs. A rough appearance of the hands was noted as well as eyelid edema. The abdominal examination was normal. There was no lymphadenopathy; no other extrapulmonary manifestations were noted. At admission, the patient had acute respiratory failure. Arterial blood gas analysis with oxygen 4 L/min showed a PaO_2_ of 50 mmHg, PaCO_2_ of 32 mmHg, pH of 7.50, and HCO_3_ of 27 mEq/L.

**Figure 1.**
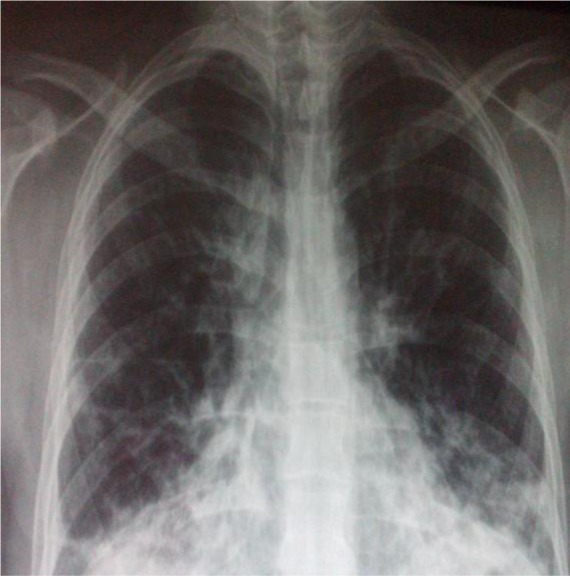
Multiple pulmonary infiltrates associated to bilateral alveolar opacities

**Figure 2.**
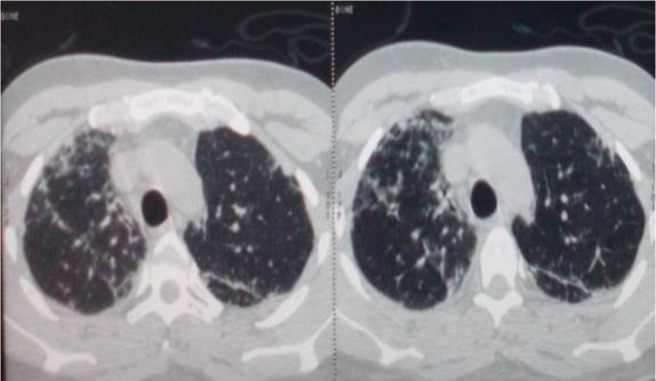
Chest High-resolution computed tomography showing bilateral micronodular opacities, traction bronchiectasis, thickening of septal lines, and a localized ground-glass opacities.

Chest radiograph showed multiple pulmonary infiltrates associated with bilateral alveolar opacities (Figure 1). Echocardiogram showed normal left ventricular function. Laboratory investigations revealed neutrophilic leukocytosis (white blood cells 12880/UL, neutrophils 10350/mL, lymphocytes 1560/mL); elevated creatine phosphokinase (CPK), 1176 U/L; elevated lactate dehydrogenase (LDH), 1193 U/L; aspartate aminotransferase (AST) level, 48 U/L (6–34 U/L); alanine transaminase (ALT) level, 29 (6–34 U/L); and C-reactive protein, 36 mg/dL (0–5 mg/dL). HIV test was negative. He was diagnosed with severe community-acquired pneumonia and treated with oxygen and intravenous corticosteroids and antibiotics (levofloxacin and cefotaxime). High-resolution computed tomography of the chest showed bilateral micronodular opacities, traction bronchiectasis, thickening of septal lines, and localized ground-glass opacities in the middle lobe and lingula (Figure 2).

On the seventh day of hospitalization, the patient’s general and respiratory conditions worsened. Since there was no evidence of bacterial, fungal, or viral infection, and owing to the increased muscle and liver enzyme values, we suspected inflammatory myopathy with ILD. Thus, we checked specific markers for connective tissue diseases. Laboratory immunological tests revealed moderately increased anti-nuclear antigen antibodies (1/100), as well as positive anti-extractable nuclear antigen (anti-Jo-1 antibodies positive and anti-nucleosome Mi2 positive); rheumatoid factor and anti-neutrophil cytoplasmic antibodies (C-ANCA and P-ANCA) were at normal values. Bronchoalveolar lavage was not performed initially. Pulmonary function tests showed a restrictive pattern on spirometry with a total lung capacity at 48% of the predicted normal value.

The diagnosis of ASS was made, and the patient continued prednisone at the dose of 50 mg/day, which was reduced gradually to 5 mg/day. Cyclophosphamide pulse therapy (750 mg. once every 45 days × 6) was started 1 month after the patient’s hospital admission. Three weeks after the first dose of Cyclophosphamide pulse, the respiratory effort had improved, and the patient was discharged without oxygen. At short-term follow-up, he reported significant improvement in his dyspnea. Patient’s respiratory condition improved (PaO_2_ 76 mmHg, PaCO_2_ 41 mmHg, pH 7.37, and HCO_3_ 24 mEq/L on room air); laboratory values for blood cell count, CPK, LDH, AST, ALT, and CRP returned to normal ranges within three weeks.

## DISCUSSION

The diagnosis of polymyositis/ dermatomyositis PM/DM-related ILD is not difficult in patients with established disease or in newly diagnosed patients with typical disease manifestations (6). However, PM/DM may not be suspected to be the cause of ILD when ILD is the only manifestation (7). Severe respiratory failure as the presenting feature of ILD associated with AAS is extremely rare (6). Acute respiratory failure is an extremely rare presentation of the ASS syndrome (6). Clinical suspicion of polymyositis is high where muscle pain or tenderness is obvious, but these symptoms are present only in 50% of the cases (8). Sub-acute polymyositis is considerably more common with progressive weakness and atrophy of proximal muscle groups. Laboratory investigation usually indicates elevated serum creatine kinase activity, which was the case with our patient. Antihistidyl-tRNA synthetase (anti-Jo-1) antibody was the first of the anti-ARS antibodies to be discovered. It is the most frequently detected antisynthetase autoantibody and is strongly associated with the presence of ILD in both PM and DM. Corticosteroids remain the cornerstone of initial empiric treatment for inflammatory myopathy (9). Among patients with antisynthetase syndrome-related ILD, the response to therapy with prednisone is heterogeneous, with 30–40% of the subjects showing improvement and 20–40% being stabilized (10, 11). Other immunosuppressive drugs should be considered at the outset of treatment, particularly in ASS and other severe and progressive manifestations of ILD (12). For patients who have responded poorly to the conventional pulse steroid therapy, increasing the intensity of pulse cyclophosphamide, cyclosporine, or other immunosuppressive therapy earlier is the best approach (13). Remission induced by the addition of an immunosuppressive drug is reported in some cases of corticosteroids resistance (14). Deaths due to ILD were rare in previous studies; mortality from respiratory failure was about 10% at a median follow-up period of 4 years (14).

This patient’s case demonstrates how the diagnosis of ASS may not be clinically evident on history or physical examination, but may become apparent with further diagnostic evaluation. Early diagnosis and appropriate treatment lead to better prognosis.

## References

[1] Tomonaga M Sakamoto N Ishimatsu Y Kakugawa T Harada T Nakashima S Comparison of pulmonary involvement between patients expressing anti-PL-7 and anti-Jo-1 antibodies. Lung 2015;193(1):79–83. 25394672 10.1007/s00408-014-9665-7

[2] Imbert-Masseau A Hamidou M Agard C Grolleau JY Chérin P. Antisynthetase syndrome. Joint Bone Spine 2003;70(3):161–8. 12814758 10.1016/s1297-319x(03)00012-5

[3] Haydour Q Wells MA McCoy SS Nelsen E Escalante P Matteson EL. Anti-synthetase syndrome presenting as cryptogenic organizing pneumonia. Respir Med Case Rep 2012;6:13–5. 26029595 10.1016/j.rmcr.2012.08.003 PMC3920447

[4] Connors GR Christopher-Stine L Oddis CV Danoff SK. Interstitial lung disease associated with the idiopathic inflammatory myopathies: what progress has been made in the past 35 years? Chest 2010;138(6):1464–74. 21138882 10.1378/chest.10-0180

[5] Solomon J Swigris JJ Brown KK. Myositis-related interstitial lung disease and antisynthetase syndrome. J Bras Pneumol 2011;37(1):100–9. 21390438 10.1590/s1806-37132011000100015 PMC3676869

[6] Piroddi IM Ferraioli G Barlascini C Castagneto C Nicolini A. Severe respiratory failure as a presenting feature of an interstitial lung disease associated with anti-synthetase syndrome (ASS). Respir Investig 2016;54(4):284–8. 10.1016/j.resinv.2016.01.005 27424829

[7] Yousem SA Gibson K Kaminski N Oddis CV Ascherman DP. The pulmonary histopathologic manifestations of the anti-Jo-1 tRNA synthetase syndrome. Mod Pathol 2010;23(6):874–80. 20228783 10.1038/modpathol.2010.65

[8] Walton J. Disorders of voluntary muscle. In: Weatherall DJ Ledingham JGG Warrell DA, eds. The Oxford Textbook of Medicine. 3rd edn. Oxford: Oxford University Press 1996; 415664158.

[9] Oddis CV. Idiopathic inflammatory myopathy: management and prognosis. Rheum Dis Clin North Am 2002;28(4):979–1001. 12506780 10.1016/s0889-857x(02)00028-5

[10] Frazier AR Miller RD. Interstitial pneumonitis in association with polymyositis and dermatomyositis. Chest 1974;65(4):403–7. 4819244 10.1378/chest.65.4.403

[11] Salmeron G Greenberg SD Lidsky MD. Polymyositis and diffuse interstitial lung disease. A review of the pulmonary histopathologic findings. Arch Intern Med 1981;141(8):1005–10. 7247586

[12] Saketkoo LA Ascherman DP Cottin V Christopher-Stine L Danoff SK Oddis CV. Interstitial Lung Disease in Idiopathic Inflammatory Myopathy. Curr Rheumatol Rev 2010;6(2):108–119. 21941374 10.2174/157339710791330740 PMC3092635

[13] Kim SH Park IN. Acute Respiratory Distress Syndrome as the Initial Clinical Manifestation of an Antisynthetase Syndrome. Tuberc Respir Dis (Seoul) 2016;79(3):188–92. 27433180 10.4046/trd.2016.79.3.188 PMC4943904

[14] Douglas WW Tazelaar HD Hartman TE Hartman RP Decker PA Schroeder DR Polymyositis-dermatomyositis-associated interstitial lung disease. Am J Respir Crit Care Med 2001;164(7):1182–5. 11673206 10.1164/ajrccm.164.7.2103110

